# Antibiotic-Releasing Porous Shell Cements

**DOI:** 10.3390/antibiotics15080781

**Published:** 2026-08-13

**Authors:** Trang N. Hau, Celine J. Agnes, Lauren E. Kemp, Walid K. Bibi, Cole C. Moore, Benjamin Levi, Antonia F. Chen, Alexander M. Tatara

**Affiliations:** 1 Division of Infectious Diseases and Geographic Medicine, The University of Texas Southwestern Medical Center, Dallas, TX 75390, USA; trang.hau@utsouthwestern.edu (T.N.H.); lauren.kemp@utsouthwestern.edu (L.E.K.); walid.bibi@utsouthwestern.edu (W.K.B.); cole.moore@utsouthwestern.edu (C.C.M.); 2 Department of Surgery, The University of Texas Southwestern Medical Center, Dallas, TX 75390, USA; benjamin.levi@utsouthwestern.edu; 3 Department of Biomedical Engineering, The University of Texas Southwestern Medical Center, Dallas, TX 75390, USA; 4 Department of Orthopedic Surgery, The University of Texas Southwestern Medical Center, Dallas, TX 75390, USA

**Keywords:** bone cement, antibiotic release, tissue engineering, osteomyelitis, biomaterials, *Staphylococcus aureus*

## Abstract

**Background/Objectives**: Solid bone cement is a poor vehicle for delivering antibiotics, and porous bone cement lacks mechanical robustness. Inspired by the different structural phases of bone, we hypothesized that porous shell cements (PSCs) could be synthesized to be both mechanically resilient and effective in eluting antibiotics. Our objective was to synthesize and characterize PSCs and compare their physicochemical properties to solid and porous cements. **Methods**: PSCs were synthesized with thin versus thick outer porous shells and compared to homogenous solid and porous cements as controls. Porosity and architecture were assessed through scanning electron microscopy and microcomputed tomography. Mechanical properties were quantified by compression testing. Antibiotic release kinetics and antibiotic activity were measured via release assays, disc diffusion assays, and minimum inhibitory concentration tests. **Results**: PSCs exhibit both solid and porous regions within a single bone cement construct with enhanced surface area to volume ratios in porous regions. Cements with porous regions have lower synthesis heat during polymerization. The mechanical strength of PSCs correlates with the ratio of solid core to porous shell thickness. The addition of the porous shell layer significantly increases antibiotic elution and increases the inhibition of *Staphylococcus aureus* and *Staphylococcus epidermidis* strains. **Conclusions**: We are able to synthesize customizable PSCs that balance the mechanical strength of solid bone cement with the antibiotic release profile of porous cement as a new biomaterial strategy to prevent orthopaedic device infection.

## 1. Introduction

With an ageing population, there is an increasing demand for permanent orthopaedic devices such as replacement joints (arthroplasty). Based on U.S. data, a 100% increase in the number of arthroplasty procedures is forecasted between 2017 and 2050 [1]. Implanted devices are at risk of periprosthetic joint infection (PJI) [2,3]. PJI incidence continues to increase despite significant infection prevention efforts [4]. In hip and knee surgeries alone, the annual cost of revision due to infection reached $566 million in 2009 [5] and is projected to reach $1.85 billion by 2030 [4,6]. The most prevalent culprits in orthopedic infection are staphylococcal species, including strains like methicillin-resistant *Staphylococcus aureus* (MRSA) and *Staphylococcus epidermidis*, which are responsible for up to 65% of cases [2,7]. Bone and device infections caused by *S. aureus* are difficult to treat due to robust biofilm production and various other immune-evasive mechanisms [7]. Therefore, strategies to prevent and treat PJI are urgently needed.

In orthopaedics, dentistry, podiatry, and other reconstructive surgeries, bone cement is used as a void-filling biomaterial. For arthroplasty specifically, bone cement is used as a “grout” when implanting a device. Bone cement is conventionally synthesized from poly(methyl methacrylate) (PMMA), a robust polymer capable of supporting weight bearing without eliciting a significant inflammatory host response. PMMA has no inherent ability to promote bone regeneration or prevent infection [8]. Antibiotics can be added prior to its polymerization for local antibiotic delivery either as prophylaxis (to prevent the development of PJI) or as part of a staged therapy to treat active PJI. However, conventional PMMA has limited antibiotic elution due to a low surface-area-to-volume ratio, with antibiotics remaining trapped within its inner matrix rather than diffusing into the local tissues [9,10]. Furthermore, the heat generated by the exothermic free radical polymerization of PMMA can damage certain antibiotics and cause loss of activity [11]. In large clinical studies, antibiotic-loaded bone cement has not been able to prevent PJI [12,13].

To improve bone cement as a drug delivery vehicle, we and others have shown that introducing porosity to PMMA through various synthetic schemes improves the delivery of antibiotics [14,15,16]. Following antibiotic elution, these pores can act as a scaffold for host tissue infiltration to promote host/biomaterial integration [17,18]. However, with the addition of porosity, PMMA inherently loses some mechanical strength, which may limit its applications in load-bearing situations. To address the challenge of preventing PJIs, there is an unmet need for cements with enhanced antibiotic elution while maintaining mechanical strength.

**Figure 1:**
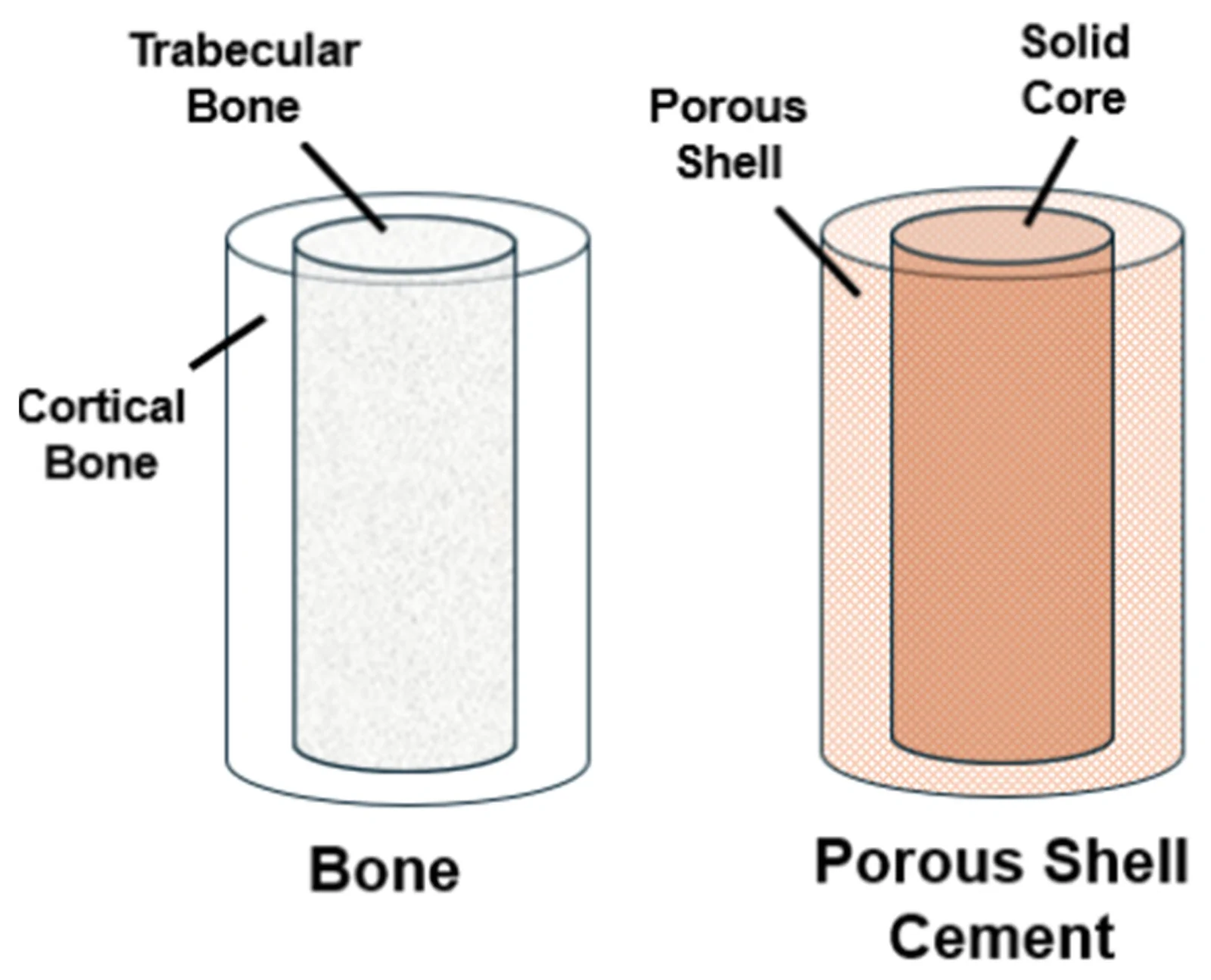
Schematic illustrating the distinct regions of bone (dense cortical bone versus porous trabecular bone) and the distinct regions of porous-shell cements.

**Figure 2:**
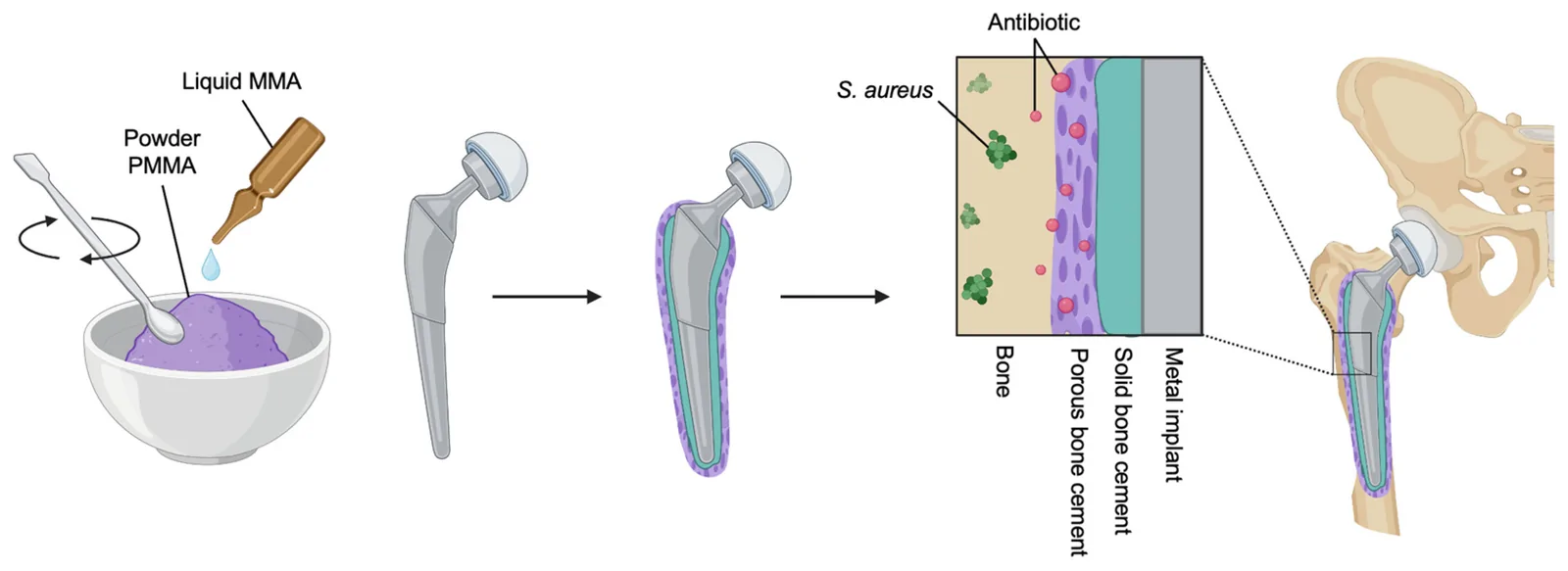
Schematic for clinical workflow for PSC fabrication and implantation. PMMA = poly(methyl methacrylate). MMA = methyl methacrylate. Created in Biorender. Kemp, L.E. (2026) https://app.biorender.com/illustrations/canvas-beta/681245bc891af6d85c11d97a (accessed on 29 June 2026).

Bone, as a tissue, faces a similar conundrum, as it must have porous space available to support hematopoietic cell populations while maintaining mechanical loads. To achieve these dual purposes, there are two distinct regions within the tissue: the porous trabeculae and dense cortex. Inspired by the regional structural differences in bone, we designed Porous Shell Cements (PSCs) as bone cements with regional differences in architecture to support different roles (Figure 1). As an inversion of bone structure due to application, PSCs have a porous outer “shell” region for antibiotic elution and a solid “core” for mechanical stability. In this study, we synthesized and characterized PSCs with different shell thicknesses to determine the tunability of PSC properties using carboxy methylcellulose (CMC) as a porogen. We measured the heat of reaction, porosity, surface-to-volume ratio, and mechanical properties (compressive strength and modulus) using single-phase solid bone cement and porous bone cement as controls. We evaluated the ability of PSCs compared to solid and porous bone cement to elute antibiotics and measured the bioactivity of released drug. Vancomycin was chosen as a model drug as it is one of the most common antibiotics employed in PMMA cements and broadly has efficacy against staphylococci, the most significant cause of PJI [9,19,20]. We hypothesized that increasing the ratio of porous shell to core would increase antibiotic delivery while decreasing mechanical strength. In clinical practice, the cement mantle thickness depends on the location and type of arthroplasty, with generally thicker cement for total hip arthroplasty (THA)—which is traditionally 2 mm—than total knee arthroplasty (TKA) [21,22]. We chose two PSC thicknesses: the thin shell PSC (0.5 mm thick) could be applied in TKA or THA whereas the thick shell PSC (1.5 mm) would be more easily applied in THA. If successful, we envision a clinical workflow where PSCs can be incorporated as part of arthroplasty implantation (Figure 2).

## 2. Results

### 2.1. Bone Cements Can Be Synthesized with Customizable Outer Porous Shells

#### 2.1.1. Cement Synthesis and Handling

**Figure 3:**
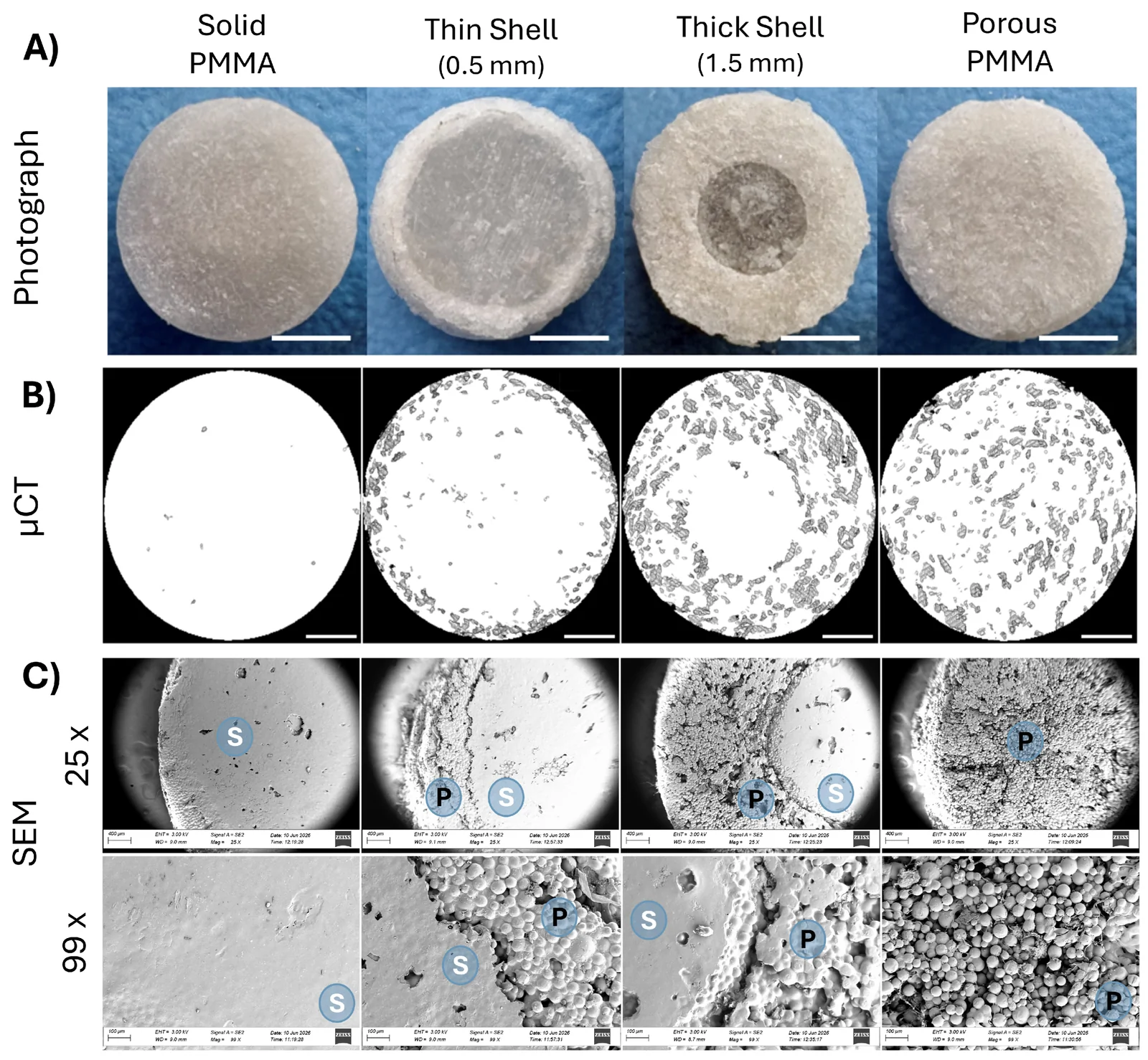
Porous-shell cements can be synthesized with adjustable porosity and shell thicknesses: (**A**) Photographs of cement discs; scale bar = 3 mm. (**B**) Microcomputed tomography surface reconstructions of cements; scale bar = 1 mm. (**C**) Representative scanning electron microscopy images of cements capturing the shell/core interface at 25× magnification (**top**); P = porous; S = solid; scale bar = 400 µm and 99× magnification (**bottom**); scale bar = 100 µm.

Cements were synthesized as 6 mm × 1 mm discs or 6 mm × 12 mm cylinders (diameter × height) for assays as recommended by field standards [23,24,25]. For PSCs, “thin” shell constructs were synthesized with a 0.5 mm thick shell and 5 mm diameter core (1:5 shell thickness: core radius ratio) and “thick” shell constructs were synthesized with a 1.5 mm thick shell and a 3 mm core (1:1 shell thickness: core radius ratio). Two distinct regions, shell and core, were easily appreciated with no obvious interfacial transition zone or gaps present (Figure 3A). Porous regions were slightly opaque compared to solid regions. Constructs could be handled without delamination between shell and core throughout all experiments.

#### 2.1.2. Heat of Synthesis

**Figure 4:**
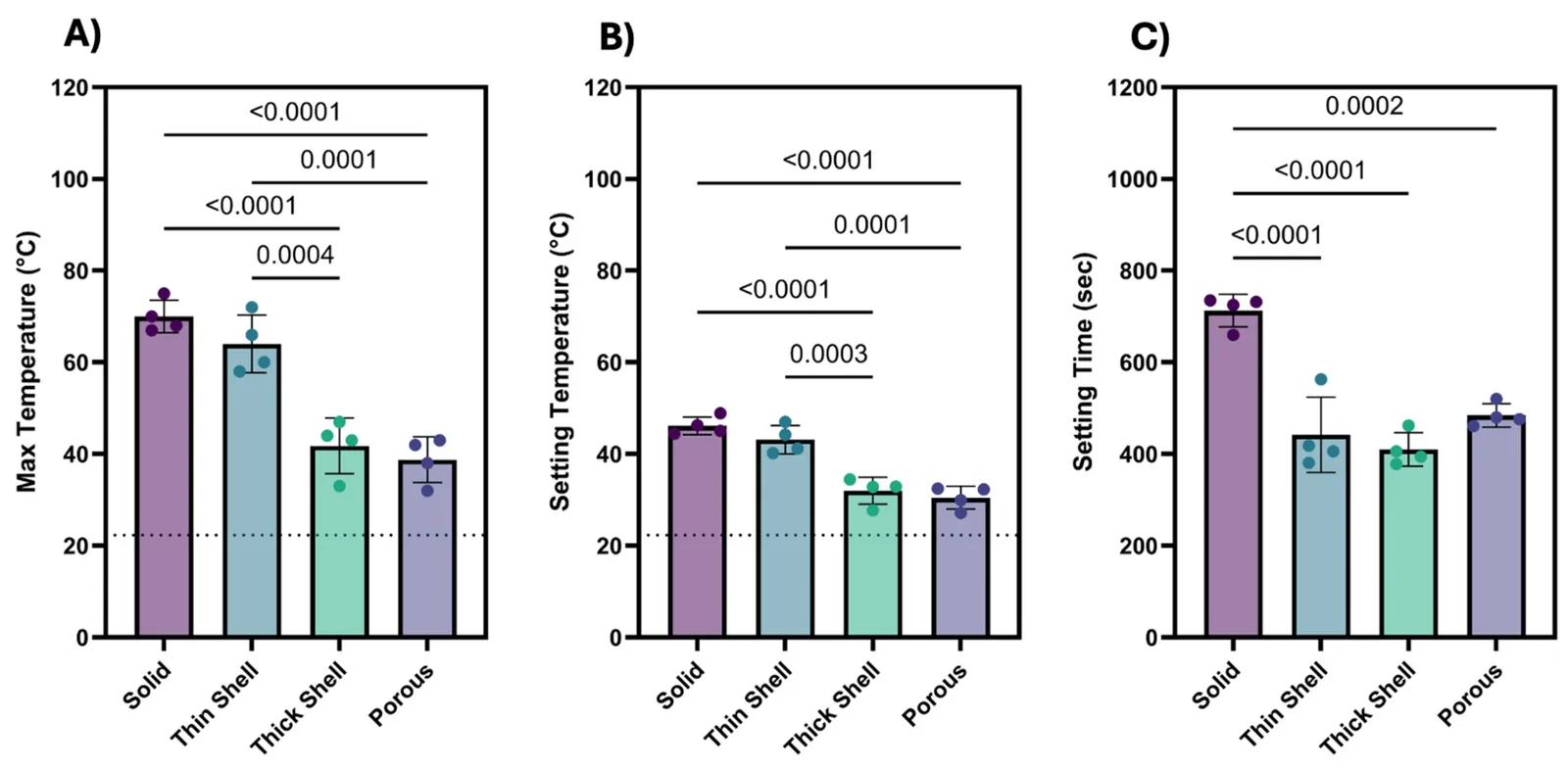
Assessment of cement synthesis. (**A**) Maximum temperature; (**B**) setting temperature; and (**C**) setting time. Dashed line indicates ambient temperature.

The maximum temperature, setting temperature, and setting time during synthesis for each group (solid, thin shell, thick shell, and porous) was measured in accordance with the International Organization for Standardization (ISO) standard 5833 (“Implants for Surgery—Acrylic resin cements”) using a thermocouple and mould/plunger setup [24]. As previously reported [15], single-phase control porous cement had a lower maximum temperature (69.98 ± 3.93 °C versus 38.63 ± 4.97 °C) and setting temperature 46.15 ± 1.96 °C versus 30.47 ± 2.49 °C) than solid cement (Figure 4). The thin shell PSCs exhibited maximum and setting temperatures of 64.00 ± 6.16 °C and 43.16 ± 3.08 °C, respectively. These values were comparable to those of the solid PMMA controls. In contrast, the thick shell PSCs demonstrated significantly lower temperatures (maximum temperature: 41.65 ± 5.89 °C, setting temperature: 31.98 ± 2.95 °C) than both the solid cement and thin shell PSCs, more similar to that of porous cement. Both PSC groups exhibited significantly shorter setting times compared with the solid cement.

#### 2.1.3. Surface Topography

Qualitatively, clear differences in the surfaces of the porous and solid layers in PSCs were observed through scanning electron microscopy (SEM) (Figure 3C). Solid regions were smoother with slight blebbing. Porous regions appeared more textured with additional divots. Images of the border area partitioning the two regions showed a distinct boundary but with no evidence of delamination during synthesis.

#### 2.1.4. Internal Architecture

**Figure 5:**
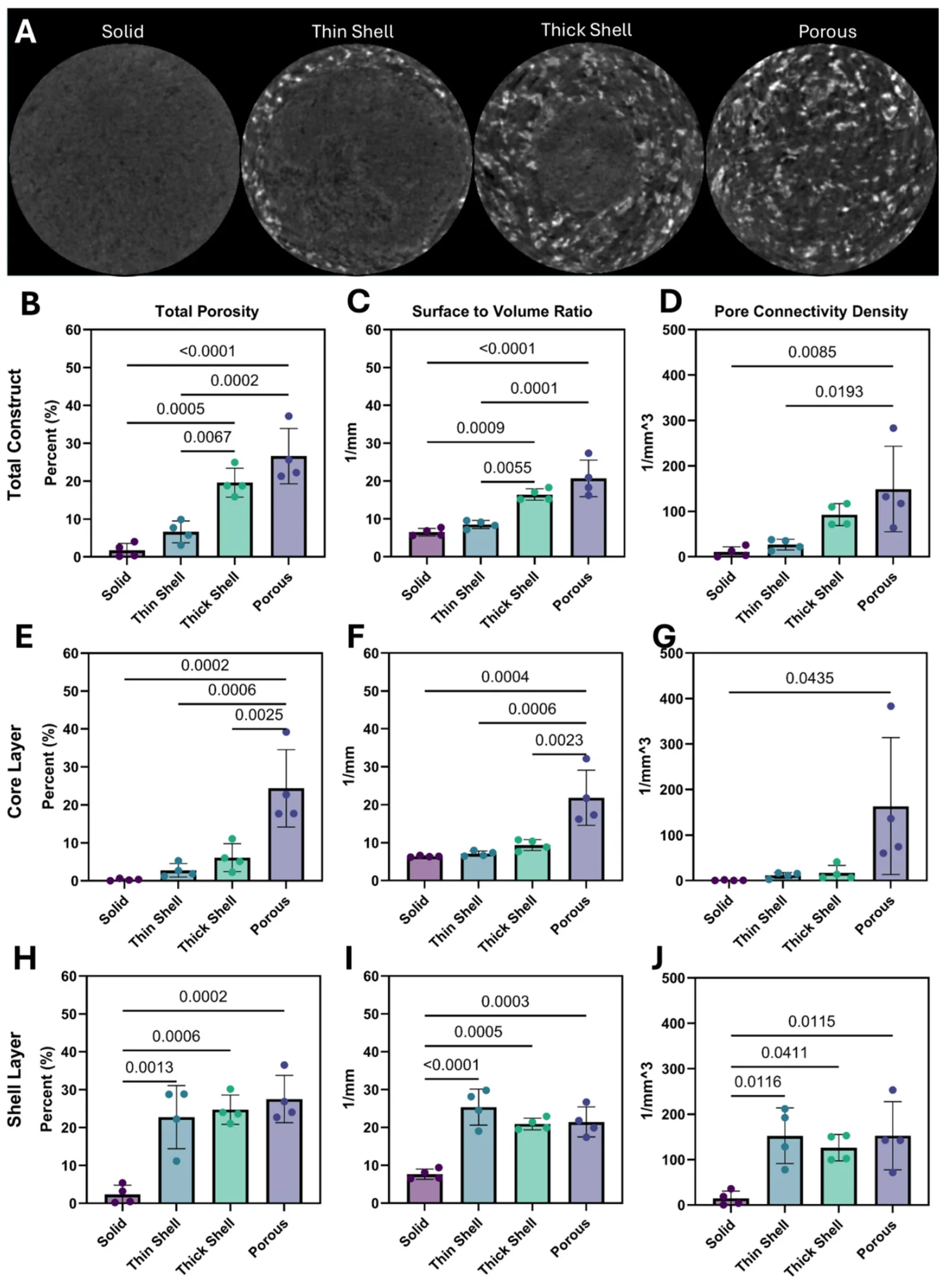
μCT assessment of structural properties for solid, porous and PSCs. Cross-sectional images of the internal construct structure. (**A**) Total porosity. (**B**,**E**,**H**) Surface-to-volume ratio. (**C**,**F**,**I**) Pore connectivity density (**D**,**G**,**J**) of constructs attained by 3D analysis for full constructs (**B**–**D**) and separated by core (**E**–**G**) and shell layers (**H**–**J**).

Microcomputed tomography (μCT) imaging revealed distinct differences in structural architecture between the control and PSC cements (Figure 3B and Figure 5). Cross-sectional images of conventional solid cement demonstrated a dense structure with a total porosity of 1.76 ± 1.83%, whereas porous samples (PMMA synthesized with 30% CMC) exhibited a markedly more textured architecture and a significantly greater total porosity of 26.60 ± 7.29% (*p* < 0.0001). Significant differences between these control groups were also observed in surface-to-volume ratio and pore connectivity density, both of which were increased in porous relative to solid cement.

The distinct regional differences in the architecture of PSCs were evident in the cross-sectional images, which demonstrated either a predominantly solid core surrounded by a thin, porous shell or a smaller solid core enclosed by a thicker porous shell. Across the entire cross-section, thin shell PSCs had an overall total porosity of 6.63 ± 2.87%. Thick shell PSCs had a significantly higher total porosity of 19.60 ± 3.81%. Surface-to-volume ratio measurements followed a similar trend, with significantly greater values observed in the thick shell constructs relative to the solid and thin shell groups. However, although pore connectivity density was increased in the thick shell constructs, it did not differ significantly from either the solid or thin shell cements (*p* = 0.1388).

To verify the structural characteristics of the individual layers (shell versus core), region-specific µCT analyses were performed. A distinct boundary separated the shell and core, with no detectable porosity gradient or interfacial transition zone. Therefore, the resulting structural parameters are representative of the intrinsic structural architecture of the core and shell, respectively. Within the core region, total porosity, surface-to-volume ratio, and pore connectivity density were comparable across the thin shell, thick shell, and solid cements, and were reduced relative to the single-phase porous cement. Conversely, analysis of the shell region for each parameter demonstrated comparable values for thin shell and thick shell PSCs to those observed in the porous group, all of which exceeded the measurements from the solid cement control.

### 2.2. PSCs Have Greater Mechanical Compressive Properties than Porous Cement

**Figure 6:**
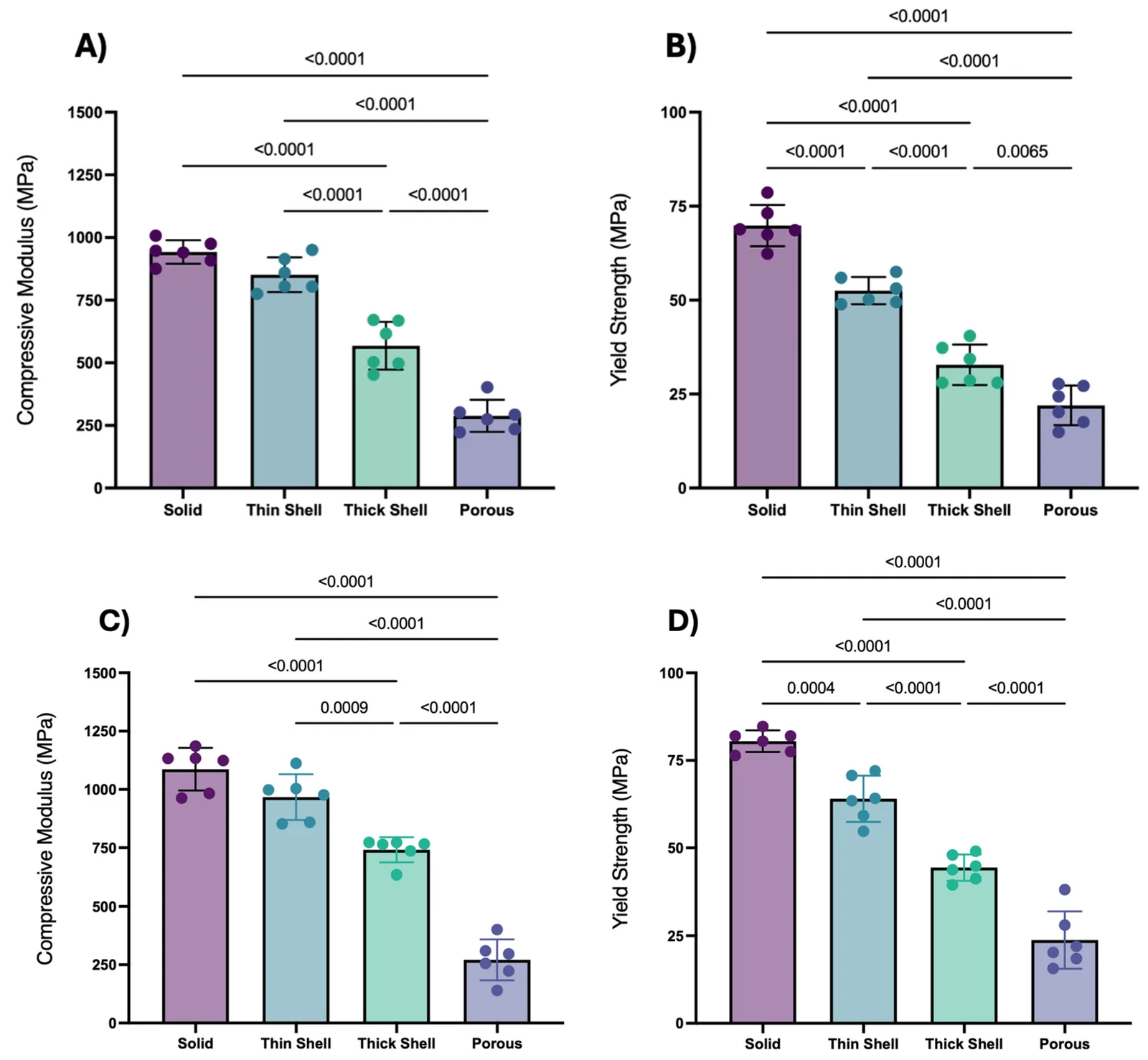
The compressive properties of cement cylinders measured per ISO 5833. (**A**) Compressive modulus and (**B**) yield strength for dental bone cement. (**C**) Compressive modulus and (**D**) yield strength for orthopaedic bone cement.

Mechanical compressive testing was performed as described in ISO 5833 on dental and orthopaedic bone cements with different formulations (PSCs and solid/porous controls) to measure the compressive modulus and 2% offset yield strength [24]. Porous cements had moduli and strengths approximately three times less than solid cements (Figure 6). Thin shell PSCs had similar moduli to solid cements and had greater moduli than thick shell PSCs and porous cements. While thick shell PSCs had approximately twice the moduli of porous cements, they were still weaker than solid cements. These trends were similar for compressive yield strength, with solid cements being strongest and PSCs being stronger than porous cement. Between the two cement types, the orthopaedic cement achieved higher compressive values and yield strength than dental resins with two of the six thin shell PSCs achieving greater than 70 MPa.

### 2.3. PSCs Deliver Antibiotics More Effectively than Solid Cement

#### 2.3.1. Encapsulation Efficiency of PSCs

**Figure 7:**
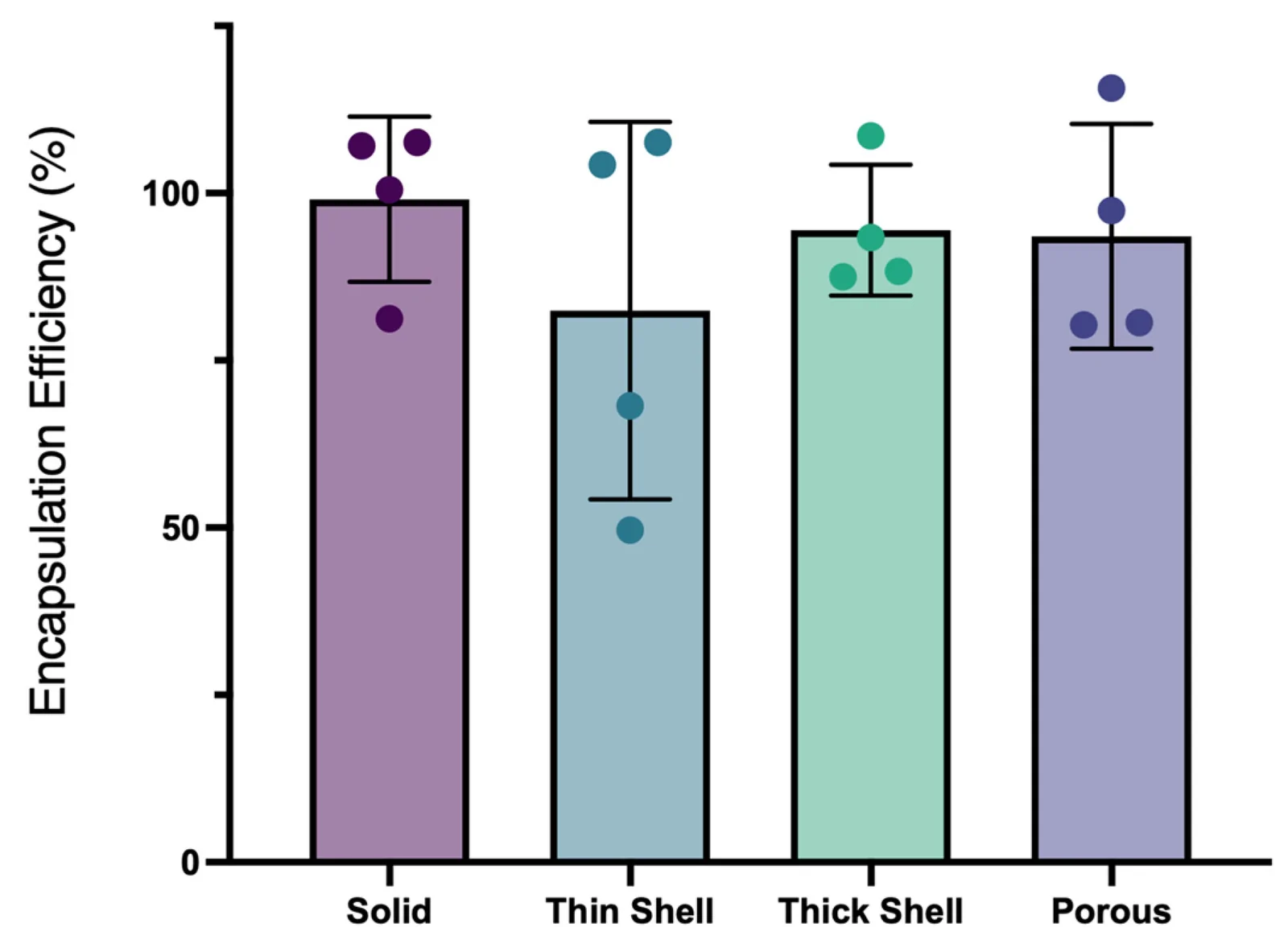
The encapsulation efficiency of vancomycin from PSCs.

To confirm the efficiency of vancomycin loading into PSC discs, an encapsulation study was performed by first degrading each disc sample in dichloromethane and deionized water then analyzing the vancomycin incorporated in each disc. All groups, regardless of porosity, were synthesized with the same concentration of vancomycin (3% wt/wt). Antibiotic loading was achieved at near 100% efficiency across groups with no significant differences (Figure 7).

#### 2.3.2. Antibiotic Elution from PSCs

**Figure 8:**
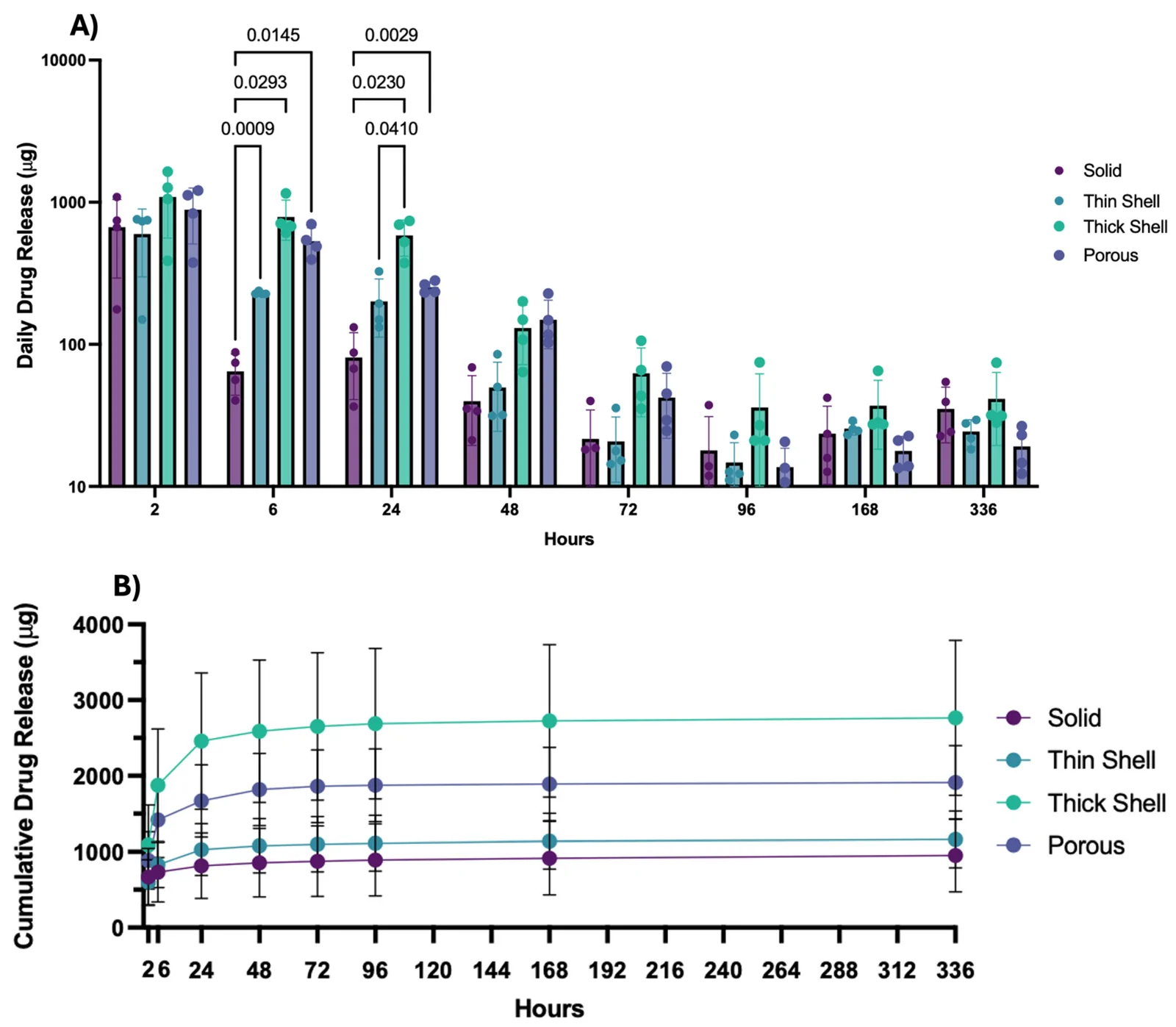
Drug delivery kinetics from vancomycin-loaded cements (3% wt/wt). (**A**) Daily drug released. Statistical analysis for daily drug release was performed with two-way ANOVA with Tukey test. (**B**) Cumulative drug release from PSC discs over 2 weeks; n = 4 per group.

To evaluate if PSCs retain the ability to effectively deliver antibiotics similar to porous cements [14,15], the concentration of antibiotic in the supernatant from vancomycin-loaded PSC discs was assayed to calculate daily and cumulative antibiotic release kinetics in an aqueous solution at 37 °C. Release kinetics exhibited a burst release across all formulations within the first 24 h (Figure 8). At early timepoints, groups containing porous regions delivered more vancomycin to the local environment. Thin shell PSCs released more vancomycin than the solid group; however, they did not differ significantly. By 336 h, thick shell PSC cumulatively released significantly more vancomycin than solid cements (*p* = 0.009) and thin shell PSCs (*p* = 0.019).

#### 2.3.3. Activity of Antibiotic Released from PSCs

Supernatants from discs were tested against three clinically relevant strains of staphylococci: methicillin-susceptible *Staphylococcus aureus* (MSSA) strain UAMS-1—a clinical isolate from human osteomyelitis; MRSA strain JE2—a commonly used derivative of the prevalent USA300 community-associated strain that is among the most common causes of MRSA osteomyelitis in the USA [26]; and methicillin-susceptible *Staphylococcus epidermidis* (MSSE) strain ATCC 14990. Minimum inhibitory concentration (MIC) testing of supernatants at different timepoints was performed per ISO 20776-1, with the first well being a 1:1 dilution of supernatant and bacterial culture [25]. Vancomycin from all cements was capable of inhibiting staphylococcal strains for at least 24 h (Table 1). Thin-shell PSCs and porous cements had consistent inhibition with as much as 8× the dilution of release vancomycin over 48 h.

#### 2.3.4. Direct Inhibition of Bacteria by Antibiotic-Loaded PSCs

**Figure 9:**
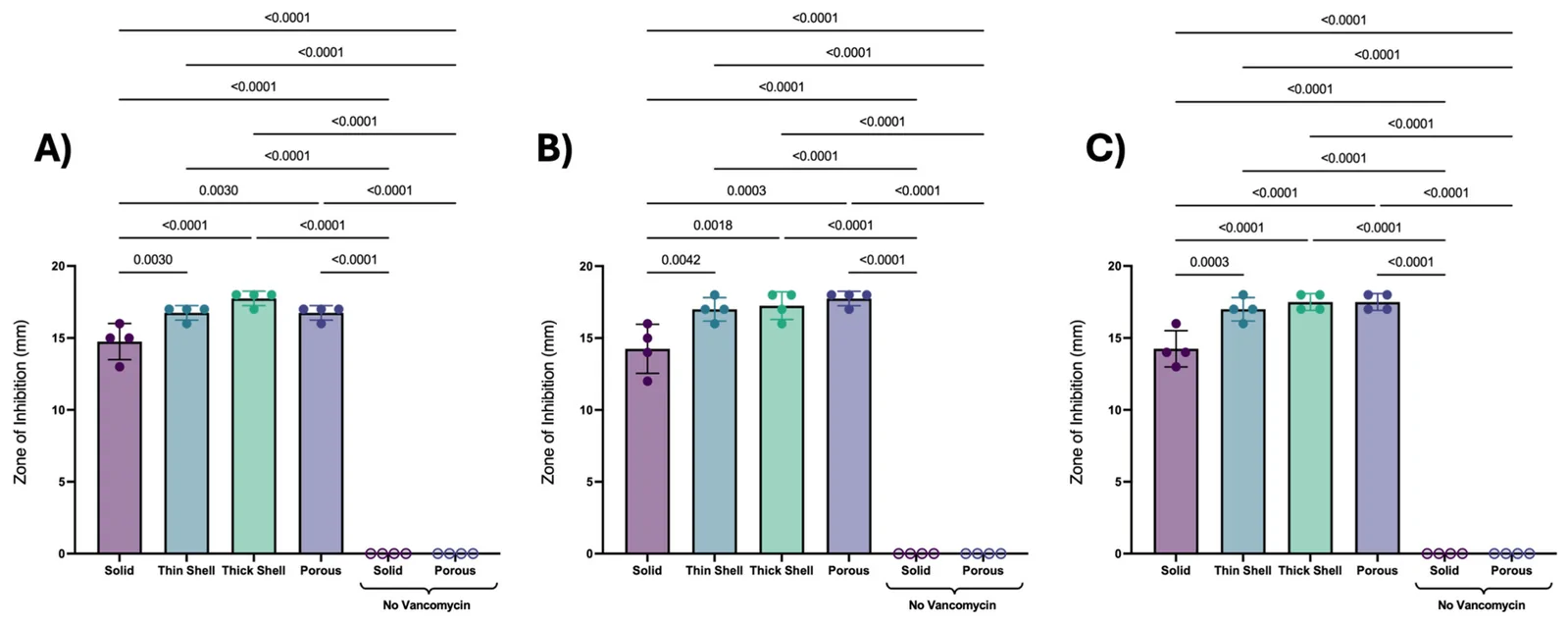
Inclusion of a porous shell confers more bacterial inhibition than solid PMMA for vancomycin-loaded cements. Standard Kirby–Bauer antibiotic disc diffusion was performed against (**A**) MSSA; (**B**) MRSA; and (**C**) MSSE; n = 4 per group.

Antibiotic-loaded cement discs were challenged against clinically relevant strains of staphylococci (MSSA, MRSA, and MSSE) in a Kirby–Bauer disc diffusion assay per Clinical & Laboratory Standards Institute (CLSI) protocol M02 [23]. Both PSC groups and the porous group generated greater zones of inhibition than solid cement against all three staphylococcal strains (Figure 9). There were no statistically significant differences in inhibition between the porous group and either of the PSCs.

## 3. Discussion

The escalating prevalence of musculoskeletal disease has placed unprecedented strain on the orthopaedic surgical infrastructure [1]. As surgical volumes continue to rise, so does the demand for sophisticated biomaterials capable of meeting the complex biological and mechanical requirements of orthopaedic reconstruction. PMMA has long served as the orthopaedic clinical standard for bone void filling and implant fixation. However, its dense structural packing serves as a double-edged sword that is mechanically advantageous but limits the elution of antibiotics. Additionally, the high exothermic temperatures generated during polymerization carry the risk of thermal necrosis of surrounding tissue and can decrease the bioactivity of released antibiotics [11]. These inherent shortcomings represent a significant and unmet gap in patient care, particularly for individuals at elevated risk of postoperative infection.

Inspired by the distinct architecture of cortical and trabecular bone, we synthesized porous shell cement (PSC), a PMMA-based biomaterial with the structural stability of solid cement and the antibiotic delivery kinetics of porous cement. Using standardized protocols [23,24,25], we characterized the synthesis temperature, structure, mechanical properties, antibiotic elution and activity of PSCs with two different shell thicknesses and compared these to conventional solid cement and homogenous single-phase porous cement.

The incorporation of CMC as a porogen into the cement constructs resulted in significantly shorter setting times compared with conventional PMMA bone cement. Consistent with previous studies, this finding may be attributed to alterations in the formulation itself, as the total mass was kept constant while the relative proportions of PMMA, liquid MMA, and CMC were adjusted. Shi et al. demonstrated that CMC-containing PMMA formulations can modify the exothermic polymerization profile, with CMC acting as an effective heat sink during cement mixing [15]. Therefore, the reduced setting times observed in our current study may similarly reflect both the incorporation of CMC and altered construct compositions, although the underlying mechanism was not directly investigated.

We demonstrated qualitatively and quantitatively via SEM and μCT that the architecture of the shell and core regions of the cement can be independently controlled (Figure 3 and Figure 5). Whether a porous region was in a thin shell, thick shell, or single-phase homogenous porous cement, the amount of porosity and the nature of that porosity (surface-area-to-volume ratio and connectivity of pores) were similar (Figure 5H–J). Since pore connectivity density reflects the interconnection of the pore network rather than porosity alone, the increased values observed for the other architectural parameters may be attributed to greater pore volumes without a corresponding significant increase in pore network connectivity. This ability to tune the porous region of the cement independent of the thickness of the region allows for design flexibility towards the goal of decoupling mechanical properties from antibiotic release kinetics.

When cement cylinders of equal diameter were compared, the stiffness (compressive modulus) of thin shell PSCs was equivalent to solid PMMA (Figure 6). While both PSC groups had significantly greater yield strength than porous PMMA, they were not equivalent to solid PMMA (thin shell PSC was 78% as strong as solid PMMA). In the context of other porous cements reported in the literature [14,27,28,29], the thin shell PSCs and thick shell PSCs lose yield strength (22% and 53%) considerably less than what would be expected based on total porosity (7% and 20%) (Table 2). However, in terms of raw values, none of the cement groups in this study (including the solid control) met the ISO 5833 minimum compressive strength of ≥70 MPa [24]. Given that PSCs had a greater percentage retention of solid PMMA strength compared to other porous cements (Table 2), thin shell PSC is considered to be the strongest composition utilizing porous PMMA. This suggests that it may more consistently fall above the 70 MPa threshold with a more robust base cement, although we were not able to demonstrate that in this study with either of the selected dental or orthopaedic backbone cements. This finding may be a limitation of hand-mixing cement; it is well-described that mixing via machine and/or vacuum achieves higher strength [30]. The scheme of layered shell PSCs can be applied whether cement is mixed by hand or by other methods, and future directions should explore the effect of mixing on mechanical properties.

Additionally, the interface between core–shell regions is not prone to delamination even when the force is applied directly to the inner core—the outer shell mechanically fails prior to delamination (Figures S1 and S2).

While there were differences in mechanical properties between thin and thick PSCs, both significantly inhibited the growth of staphylococci when combined with vancomycin, including MRSA, as effectively as porous cement and significantly more effectively than solid cement (Figure 8). Although PSCs have localized similarities due to the same solid and porous layers, we hypothesize that the interconnected architecture and increased surface-to-volume ratio from integration of CMC around the PMMA network (Figure 5) likely facilitates increased and rapid antibiotic diffusion on a macroscale (Figure 8). In addition, the decreased heat of synthesis in groups with porosity may further preserve the bioactivity of released antibiotic (Figure 4, Table 1). While the thicker shell continued to release antibiotic longer than the other groups (Table 1), there were no differences in direct bacterial inhibition between thin, thick, and entirely porous cements (Figure 9). This suggests that burst release from all three of these formulations may be sufficient to prevent biomaterial colonization when exposed to bacteria at early timepoints, even as antibiotic release tapers off beyond 72 h.

With enhanced antibiotic delivery, albeit the trade-off of mechanical strength, PSCs may find use as a local antibiotic prophylaxis for TKA and THA or as non-load-bearing spacers for PJI treatment, although they will likely require continued refinement for use in load-bearing indications. Thick shell PSCs may not have capacity for load-bearing but may generate interest in other applications such as void fillers, non-weight-bearing spacers, or antibiotic-releasing beads, as PSCs are efficient in drug loading capabilities and can deliver active doses of antibiotic more localized and effectively (Figure 7 and Figure 8) [10,31,32,33]. In terms of translating PSCs into clinical practice, PSCs can be prepared in the operating room on demand or as prefabricated arthroplasty devices pre-loaded with antibiotics (Figure 2). Given their lower heat of synthesis, PSCs would be a good candidate for a variety of antibiotic agents and may even be able to support drug delivery for other classes of therapeutics, such as osteogenic growth factors, that previously could not be delivered from bone cements due to harsh synthesis condition.

Strengths of this study include the use of validated industry-standard protocols (ISO 5833, ISO 20776-1, and CLSI M02 [23,24,25]), the evaluation of two different shell configurations, the testing against three bacterial strains including MRSA, and the use of biomaterials that are currently components in US Food and Drug Administration-approved products/indications. The porogen used in this study, CMC, is widely used in foods, cosmetics, and pharmaceuticals as a safe thickening agent [34]. Porous cements derived from PMMA and CMC have been proven to be non-toxic in a variety of preclinical models as well as a case series of human patients [35,36,37]—this is encouraging for the biocompatibility of PSCs, which use even less CMC than fully porous cements. Limitations include the use of base cements with intrinsically lower mechanical properties and a lack of mechanical characterization over time experiments (such as with cyclical stress/fatigue), which is outside of the scope of ISO 5833. Pore architecture was radiographically defined, but methods such as mercury porosimetry/Brunauer–Emmett–Teller (BET) analysis could provide additional insights and validate radiographic findings on the PMMA network in future work. Our study is limited to a single antibiotic (vancomycin), an agent effective for the staphylococci that most commonly cause orthopaedic infections. Most clinically available antibiotic-loaded cements contain an aminoglycoside [38,39,40], and future work can focus on head-to-head comparisons of PSCs loaded with aminoglycosides. Further studies on the efficacy of antibiotic release on mature biofilm would be beneficial to further explore PSCs for therapy rather than prophylaxis. Additionally, we did not formally study the effects of sterilization via ethylene oxide on PSCs. Finally, this was an in vitro study and cannot fully recapitulate the complex conditions appreciated in vivo.

Regarding future directions, the findings of this study highlight several promising avenues for the continued development of next-generation bone cements. Future iterations of this platform could prioritize expanding on geometric versatility, including multilayered porous/solid/porous configurations to further refine the tunability of mechanical and biological properties for patient-specific applications. CMC is an inert porogen; future PSCs could leverage other porogens that have bioactivity against pathogens, such as medical-grade honey or chitosan [14,28]. In this study, porous regions were fixed at 30% porosity to balance porosity and elution mechanics with mechanical properties and delayed MMA release, as well as for comparison to other studies that have used 30% porogen in bone cement including CMC [14,29]. Different formulations of CMC can be tuned by the amount of porogen added [14,27,29] in order to further explore the decoupling of mechanical and drug release parameters. Single-phase homogenous porous bone cements have been demonstrated to act as cell scaffolds to promote tissue infiltration at the biomaterial/host interface [17,18], and future work will study the ability of PSCs to function as a cell microenvironment in the porous phase of PSCs in vitro after antibiotic release and in preclinical models of arthroplasty with and without infection [41,42]. Since bacterial inhibition from antibiotic release follows burst kinetics, subsequent tissue infiltration would not impact the antimicrobial benefits of PSCs. Collectively, these directions chart a course toward a multifunctional bone cement scaffold that simultaneously addresses the mechanical, regenerative, and infectious challenges of traditional bone cement in orthopedic surgery.

## 4. Materials and Methods

### 4.1. Synthesis of Porous-Shell Cements (PSCs)

The 9% (*w*/*v*) CMC stock was prepared by manually mixing powdered low viscosity CMC (MilliporeSigma, Darmstadt, Germany; 250,000 g/mol) with deionized water. The mixture was allowed to rehydrate overnight at 4 °C and mixed again to form a uniform paste. The 9% CMC stock was stored at 4 °C until use.

Bone cement synthesis was performed using a 2:1 ratio of powder weight to liquid volume from a PMMA kit. Dental cement (Great Lakes Dental Technologies, Tonawanda, NY, USA) kits containing powder PMMA polymer and liquid methyl methacrylate (MMA) monomer with a 2% (wt/wt) Dimethyl-Toluidine were used. All dental cement constructs were loaded with 7.5% (wt/wt) barium sulfate (Thermo Fisher Scientific, Waltham, MA, USA) as a radio-opacifier and 3% (wt/wt) vancomycin (Thermo Fisher Scientific, Waltham, MA, USA).

For one experiment (Figure 6), an alternative orthopaedic cement was used. Surgical Simplex P Radiopaque Bone Cement (Stryker, Chicago, IL, USA) containing the powder polymer PMMA component of 75% PMMA-Styrene co-polymer, 15% PMMA, 10% barium sulfate and 1.7% benzoyl peroxide and liquid monomer MMA with 2.6% toluidine and 75 ± 15 ppm hydroquinone. Orthopaedic cement constructs were loaded with 3% (wt/wt) vancomycin (Thermo Fisher Scientific, Waltham, MA, USA).

All powder components were weighed and combined into a 25 mL glass beaker. For porous constructs, the 9% CMC gel was added for a 30% (wt/wt) ratio with bone cement components into the beaker and premixed with the powdered components before the liquid MMA was added. Upon adding liquid MMA, the components were mixed with a metal spatula for 30 s until a dough was formed. The dough was transferred and pressed into polytetrafluoroethylene moulds with glass covers to produce either 6 mm (diameter) by 1 mm (height) discs or 6 mm (diameter) by 12 mm (height) cylinders based on Clinical and Laboratory Standards Institute and International Organization for Standardization (ISO) parameters [23,24,25]. PSCs were synthesized with the outer porous layer first. After 5 min of initial curing, cores were removed from the discs with 3 mm and 5 mm biopsy punches (Fisher Scientific, Waltham, MA, USA) or thick and thin shells, respectively. Shell cylinders were pressed with an interconnecting lid with rods of the respective diameters after the same drying time. Porous shells were given 15 min to partially cure and then filled with a solid PMMA core. Constructs were left to cure overnight before being removed and buffed of any excess with 150 grit sandpaper (Norton, Tempe, AZ, USA) and stored at 4 °C until use. Preparation of replicates were 6 per group for cylinders and 4 per group for discs per assay as specified by ISO 5833 and CLSI M02. Discs were loaded into permeable sterilization bags (Fisherbrand, Pittsburgh, PA, USA) and underwent ethylene oxide sterilization at ambient temperature with a 12 h terminal sterilization step followed by a 2 h aeration step (Anprolene AN76j, Haw River, NC, USA) before antibiotic elution and disc diffusion assays.

Discs for imaging (μCT or SEM) and cylinders for mechanical testing underwent porogen leaching in 40 mL of deionized water for 48 h under mild shaking conditions at room temperature before freezing for 8 h at −80 °C then lyophilization for 48 h in a FreeZone freeze-dryer (Labconco, Fort Scott, KS, USA) to ensure removal of any excess liquid.

### 4.2. Maximum Temperature, Setting Temperature and Setting Time Measurements

A mould and plunger, both fabricated from polytetrafluoroethylene (PTFE), were manufactured according to the dimensions specified in ISO 5833 [24]. To obtain temperature measurements, a thermocouple wire was inserted into the mould through a dedicated channel located at the centre of the die, with the probe positioned just above the internal surface of the mould. The thermocouple was connected to a datalogger and maintained at ambient room temperature prior to starting the experiments, with the ambient temperature recorded for subsequent calculations.

For all groups, 25 g of total components were used, with varying ratios of CMC, PMMA, liquid MMA, and barium sulfate depending on the intended group formulation. In the PSC groups, the core and shell layers could not be measured separately. Therefore, the ratio of core volume to total construct volume was calculated and subsequently used to determine the composition and appropriate ratios of components for each cement formulation.

The solid components were weighed out and thoroughly mixed prior to the addition of the liquid MMA component. Recording on the datalogger commenced immediately following the addition of the liquid component. Once transferred into the mould, the plunger was inserted to seal the system and c-clamps were applied to secure the plunger and mould. Temperature recording continued until the maximum temperature had been reached and a subsequent decline in temperature was observed.

The setting temperature was calculated using Equation (1). The setting time was defined as the duration from the onset of mixing with the MMA liquid component until the cement reached the calculated setting temperature [24].

(1) $$T_{set}=\frac{T_{max}+T_{amb}}{2}$$

### 4.3. Scanning Electron Microscopy (SEM)

Leached and lyophilized discs were sputter-coated with 2 nm of gold and palladium at 30 mA with an EM ACE600 sputter coater (Leica, Wetzlar, Germany) and immediately imaged in a Sigma VP field emission scanning electron microscope (Zeiss, Oberkochen, Germany) at an accelerating voltage of 3 kV. Images were taken at a general magnification of 25× and a large magnification of 99×, specifically targeting the shell/core interface of the PSCs.

### 4.4. Microcomputed Tomography (μCT)

Cement discs (n = 4 per group) were scanned using a Skyscan 1272 High-Resolution μCT (Bruker, Karlsruhe, Germany) to quantify internal porosity and connectivity. Scanning parameters were voltage of 80 kV, current of 124 µA, 1k resolution, isotropic voxel size of 17.1 µm, rotation step of 0.2°, frame averaging of 3, and no random movement correction as reported previously [14]. Acquired images were reconstructed using NRecon (Bruker), with a ring artifact reduction of 5, no smoothing, and a beam-hardening correction of 15. The reconstructed datasets were aligned using Dataviewer software (Bruker, version 1.7.0.1) prior to generating a 25-slice (0.311 mm) volume of interest (VOI) in CT analyser (CTAn, Bruker, version 1.23.0.1). The VOI was further restricted to a circular region measuring 5.3865 mm in diameter to exclude edge artifacts from the constructs.

For shell groups, sizing of the core VOIs was determined by hand measuring several representative samples, followed by estimating core diameters of approximately 4.822 mm and 2.8215 mm diameter for the thin- and thick-shell constructs, respectively. Core VOIs were processed using the custom processing tool by applying thresholding (34 to 76), despeckling to remove black speckles smaller than 10 pixels, and subsequent 3D analysis. Shell VOIs were generated using the subtractive VOI tool to include all regions outside the core VOI. Because the constructs’ cross-sections were circular, while the reconstructed images in the software were square, additional thresholding (2-255), a closing operation (radius: 25), and an ROI shrink-wrap function were applied to define the appropriate region of interest for further analysis. Shell VOI datasets were then reloaded and processed using the same procedure as for the core VOIs. Total porosity and connectivity density values were obtained directly from the 3D analysis outputs generated using Bruker CTAn software. Connectivity density was calculated by normalizing the connectivity value by the tissue volume. The connectivity parameter was determined by the software using the ConnEuler method based on the Euler characteristic approach [43].

### 4.5. Mechanical Characterization

Mechanical testing was performed on leached and lyophilized cylinder constructs using a TA HD Mechanical Testing System (Texture Technologies Corp., South Hamilton, MA, USA). Compression moduli and strength were evaluated per ISO 5833 parameters [24]. Briefly, compression force at a crosshead speed of 20 mm/minute was applied until the construct underwent fracture or surpassed the upper yield point (n = 6 per group). The slope of force over machine displacement was measured between the tare force and yield force to calculate the modulus. Compression properties were measured using Exponent Connect (Texture Technologies Corp.).

### 4.6. Encapsulation Efficiency

Discs (n = 4 per group) were weighed and placed in individual 20 mL scintillation glass vials (Fisherbrand) with 5 mL of dichloromethane (Sigma Alrich, St. Louis, MO, USA). Samples were shaken for 30 min at 300 rpm and shaken for 15 min at 300 rpm after the addition of 5 mL of deionized water. Samples were centrifuged at 4550 rpm for 5 min at room temperature to separate the organic phase. The aqueous phase was transferred to a 15 mL conical tube (Falcon, Corning, NY, USA) and stored at −20 °C until μBCA analysis. The calculation of total drug added per disc was based off initial disc weights.

### 4.7. Antibiotic Elution

Individual sterile discs (n = 4 per group) were submerged in 1 mL of sterile phosphate-buffered saline (PBS; Corning, Corning, NY, USA) and incubated at 200 rpm at 37 °C under gentle agitation. Supernatant was collected at 2, 6, 24, 48, 72, 96, 168, and 336 h time points and stored at −20 °C until analysis. After collection, supernatant was replaced with 1 mL of fresh sterile PBS.

### 4.8. μBCA (Micro Bicinchoninic Acid)

Once all timepoints were collected from the antibiotic elution step, a μBCA Protein Assay Kit (APExBIOTechnology, Houston, TX, USA) was used to determine the concentration of vancomycin, as performed in other studies [44,45]. A μBCA assay is based on the reduction of Cu^2+^ ions to Cu^+^, followed by the formation of a purple bicinchoninic acid (BCA)-Cu^+^ complex that is proportional to analyte concentration. Vancomycin can also reduce Cu^2+^ due to its phenolic functional groups. Briefly, a 2 mg/mL vancomycin stock solution in sterile PBS was diluted according to the manufacturer’s protocol to achieve a standard curve of known concentrations ranging from 200, 40, 20, 10, 5, 2, and 0 µg/mL. Then, 150 μL of each standard curve solution (n = 2 per concentration) and supernatant were placed into its own well in a 96-well plate and inoculated with 150 μL of working buffer. The plate was incubated at 37 °C for 2 h. Following incubation, the plate was then read using a CLARIOstar Plus plate reader (BMG LabTech, Ortenberg, Germany) at 562 nm. From the absorbance readings, the vancomycin concentration of each sample was calculated against the average value of the standard curves with confirmation of a linearity value (r^2^) surpassing of 0.99.

To quantify the concentration of antibiotic encapsulated following complete degradation of disc samples, a μBCA was performed against the same standard curve concentrations as stated above for disc elution. As total antibiotic encapsulation yields a higher concentration than general elution, each sample was measured directly from stock, then serially diluted two-fold to fit within range of the standard curve.

### 4.9. Antimicrobial Characterization

The minimum inhibitory concentration (MIC) of vancomycin supernatant released from PSCs was assayed on a 96-well plate. *Staphylococcus* cultures were grown at 37 °C at 200 rpm in tryptic soy broth (TSB; BD Biosciences, San Jose, CA, USA). Fresh cultures of JE2 were grown for 2–3 h and diluted to 0.5 McFarland for MIC testing. Released supernatants collected in the antibiotic elution section were thawed from −20 °C at room temperature and vortexed. Supernatant was serially diluted until a 1:512 dilution, with sterility and growth controls included for each plate. A 0.5 McFarland JE2 culture was added to supernatant dilutions at a 1:1 ratios and incubated for 18–24 h per ISO 20776-1 [25]. For each timepoint, the well at the lowest dilution without bacterial growth (i.e., containing a concentration of antibiotic high enough to inhibit growth) was recorded.

MSSA strain UAMS-1, MRSA strain JE2, and *S. epidermidis* strain ATCC 14990 (MSSE) were selected for Kirby–Bauer disc diffusion assays as bacterial species commonly associated with orthopedic infection [2]. *S. aureus* strains were generously provided by Dr. David Greenberg of UT Southwestern Medical Center. Cultures were grown for 2–3 h to log phase from overnight cultures in TSB. Fresh cultures were diluted to 0.5 McFarland and streaked onto 5% sheep blood agar plates (Thermo Fisher Scientific, Waltham, MA, USA) using sterile cotton swabs. Sterile discs were transferred with forceps onto plates and incubated for 18–24 h at 37 °C per CLSI protocol M02 [23]. The diameters of zones of inhibition were averaged between two measurements and rounded to the nearest millimetre.

### 4.10. Strength of Delamination

Delamination testing was performed on leached and lyophilized cylinder constructs using a TA HD Mechanical Testing System (Texture Technologies Corp.). Maximum force was evaluated until mechanical failure or 12 mm of displacement from the starting position. Briefly, compression force with a probe of 2.35 mm diameter challenged cements held in place by a width-adjusted rig at the PSC interface. A crosshead speed of 20 mm/minute was applied until the construct underwent delamination, other mechanical failure, or surpassed the 12 mm distance allowance (n = 3 per group). Force was measured using Exponent Connect (Texture Technologies Corp.).

### 4.11. Statistical Analysis

Statistical analysis was conducted by one-way ANOVA with post hoc analysis by the Tukey honestly significant difference test using Graph Pad Prism (version 11.0.0.) unless otherwise stated. *p*-values are reported when under the 0.05 threshold for statistical significance, and error bars are included to represent standard deviation.

## 5. Conclusions

The stagnation of bone cement development in the context of a rapidly evolving infectious landscape presents a critical vulnerability in orthopedic care that demands innovation at the intersection of materials science and microbiology. In this study, we synthesized and characterized an antimicrobial bone cement inspired by the distinct structures of bone tissue. Porous-shell cements allow for enhanced antibiotic delivery while minimizing decreases to cement mechanical stability. Future work will focus on performance in preclinical models of periprosthetic joint infection.

## 6. Patents

The Board of Regents of the University of Texas on behalf of UT Southwestern Medical Center has a patent pending on this technology.

## Figures and Tables

**Table 1:** Vancomycin release from PSCs maintains bioactivity against staphylococcal species for at least 48 h. The number in each cell indicates the number of well dilutions (2× per well) with inhibited bacterial growth (n = 4 per group). Red indicates fewer wells inhibited and blue indicates more wells inhibited, while white represents intermediate numbers of wells inhibited.

Strain	Group	2 Hrs	6 Hrs	24 Hrs	48 Hrs	72 Hrs	96 Hrs	168 Hrs	336 Hrs
***S. aureus* UAMS-1**	Solid	5.75	1.75	1.25	0.25	0	0	0	0
	Thin	4.5	4	3.5	0.75	0	0	0	0
	Thick	6	6.25	5.75	3	1	0.25	0.25	0.25
	Porous	6.5	5.5	5.75	3.5	0.5	0	0	0
***S. aureus* JE2**	Solid	5.5	1.75	1.75	0.25	0	0	0	0
	Thin	5	4	3.5	1.25	0.25	0	0	0
	Thick	6	6	5.25	3	1.5	0.5	0.25	0
	Porous	5.5	5.5	5.75	3.5	0.75	0	0	0
***S. epidermidis***	Solid	4.25	1.75	1.5	0.25	0.25	0	0	0
	Thin	4.5	3.75	3	0.75	0	0	0	0
	Thick	5.75	5.25	5.5	3	0.75	0.25	0.25	0
	Porous	5.75	4.75	5.75	3.25	0.5	0	0	0

**Table 2:** Comparison of PSC yield strength to porous cements reported in the literature.

**Author**	**Porogen**	**Total Porosity (%)**	**Loss of Yield Strength Relative to Solid (%)**
Boger et al. [27]	Hydroxypropylmethyl cellulose	10%	~33%
Kemp et al. [14]	Medical-grade Honey	13%	38%
Han et al. [28]	Chitosan	13%	~60%
Wang et al. [29]	Carboxymethyl cellulose	17%	~69%
Boger et al. [27]	Hydroxypropylmethyl cellulose	20%	~50%
Han et al. [28]	Chitosan	21%	~44%
Kemp et al. [14]	Medical-grade Honey	27%	72%
**Current Study**	**Group**	**Total Porosity (%)**	**Loss of Yield Strength Relative to Solid (%)**
Hau et al.	Thin-shell PSC	7%	22%
Hau et al.	Thick-shell PSC	20%	53%
Hau et al.	Porous	27%	84%

## Data Availability

Following publication, raw data associated with this manuscript will be uploaded to the UT Southwestern Research Data Repository (https://doi.org/10.18738/T8/5VM1QJ).

## Supplementary Materials

The following supporting information can be downloaded at: https://www.mdpi.com/article/10.3390/antibiotics15080781/s1, Figure S1: Maximum Force of Delamination Assay; Figure S2: Representative Images for Delamination Assay on PSCs. (A) Schematic of Delamination Assay; (B) Thick PSC after Delamination Assay with Intact Core–Shell Interface.

## Abbreviations

The following abbreviations are used in this manuscript:

CMC	Carboxymethylcellulose
BET	Brunauer–Emmett–Teller
DCM	Dichloromethane
ISO	International Organization for Standardization
MIC	Minimum Inhibitory Concentration
MRSA	Methicillin-Resistant *Staphylococcus aureus*
MRSE	Methicillin-Susceptible *Staphylococcus epidermidis*
MSSA	Methicillin-Susceptible *Staphylococcus aureus*
MSC	Mesenchymal Stromal Cells
PBS	Phosphate-Buffered Saline
PMMA	Poly (Methyl Methacrylate)
PSC	Porous Shell Cement
SEM	Scanning Electron Microscopy
TSB	Tryptic Soy Broth
VOI	Volume of Interest
μBCA	Micro Bicinchoninic Acid
μCT	Microcomputed Tomography
