# Supplementary material for: Antibiotic-Releasing Porous Shell Cements
Source: Antibiotics (Basel). 2026 Aug 13;15(8):781. doi: 10.3390/antibiotics15080781 (PMC13510124; doi:10.3390/antibiotics15080781)
Supplement: Supplementary file 1 [file antibiotics-15-00781-s001.zip › antibiotics-4436467-supplementary.pdf]

SUPPLEMENTAL DATA

Manuscript Title: Antibiotic-Releasing Porous Shell Cements

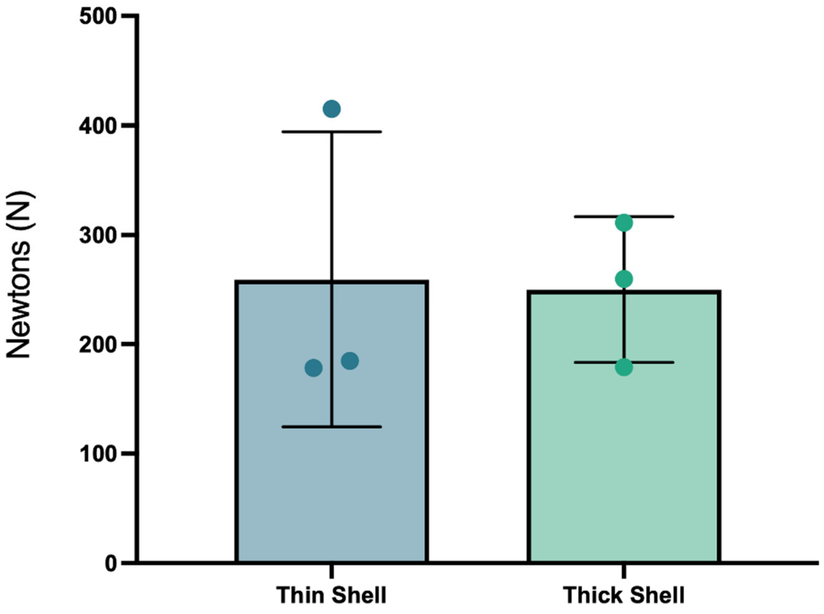

Figure S1. Maximum Force of Delamination Assay.

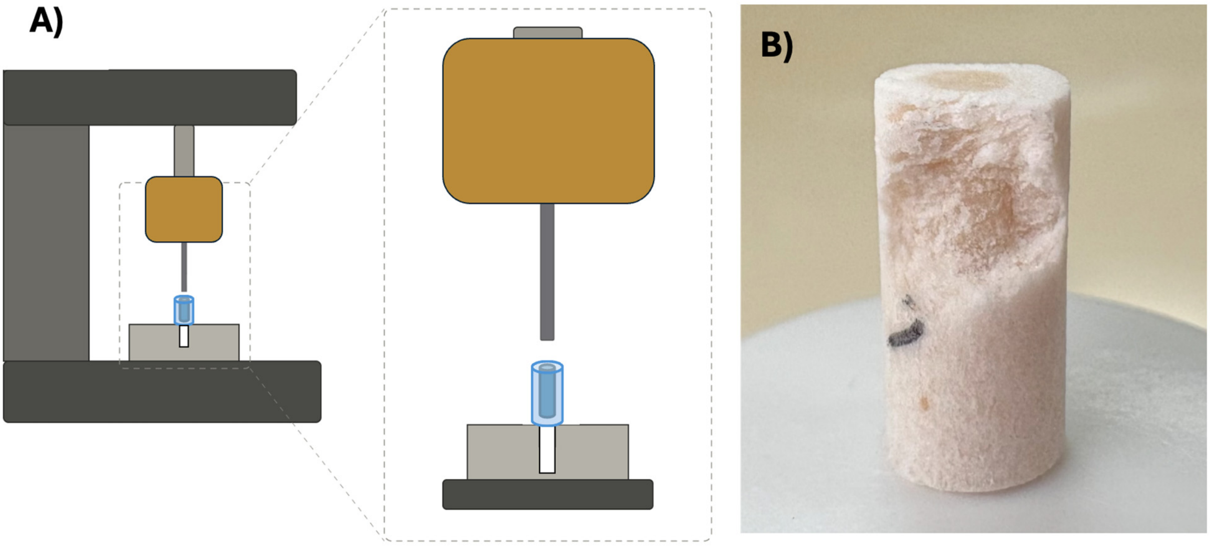

Figure S2. Representative Images for Delamination Assay on PSCs. (**A**) Schematic of Delamination Assay; (**B**) Thick PSC after Delamination Assay with Intact Core–Shell Interface.
